# Bioinspired morphing wings for extended flight envelope and roll control of small drones

**DOI:** 10.1098/rsfs.2016.0092

**Published:** 2017-02-06

**Authors:** M. Di Luca, S. Mintchev, G. Heitz, F. Noca, D. Floreano

**Affiliations:** 1 School of Engineering, Brown University, Providence, RI, USA; 2 Laboratory of Intelligent Systems, École Polytechnique Fédérale de Lausanne, Lausanne, Switzerland; 3 HEPIA (University of Applied Sciences – Western Switzerland), Geneva, Switzerland

**Keywords:** morphing wing, micro air vehicles, bioinspired drone, bioinspired aerodynamics, feathered wing

## Abstract

Small-winged drones can face highly varied aerodynamic requirements, such as high manoeuvrability for flight among obstacles and high wind resistance for constant ground speed against strong headwinds that cannot all be optimally addressed by a single aerodynamic profile. Several bird species solve this problem by changing the shape of their wings to adapt to the different aerodynamic requirements. Here, we describe a novel morphing wing design composed of artificial feathers that can rapidly modify its geometry to fulfil different aerodynamic requirements. We show that a fully deployed configuration enhances manoeuvrability while a folded configuration offers low drag at high speeds and is beneficial in strong headwinds. We also show that asymmetric folding of the wings can be used for roll control of the drone. The aerodynamic performance of the morphing wing is characterized in simulations, in wind tunnel measurements and validated in outdoor flights with a small drone.

## 1. Introduction

Morphing wings that change the shape and configuration of an aircraft can expand the flight capabilities of a flying vehicle to fulfil opposing requirements [1]. This capability is particularly important for small drones, also known as micro air vehicles (MAVs), that can navigate in close proximity to obstacles. These MAVs should be highly manoeuvrable in order to rapidly change course with a small turn radius: for a given weight of the aerial vehicle, a small turn radius is obtained by maximizing the wing surface and the lift coefficient of the wing [2]. However, wings with a large surface are very sensitive to wind; while, wings with a small surface generate less frictional drag allowing an aerial vehicle to fly faster and keep a constant forward ground speed in comparatively stronger headwinds. A wing with a morphing surface could adapt its aerial surface to optimize aerodynamic performance to specific flight situations.

**Figure 1.**
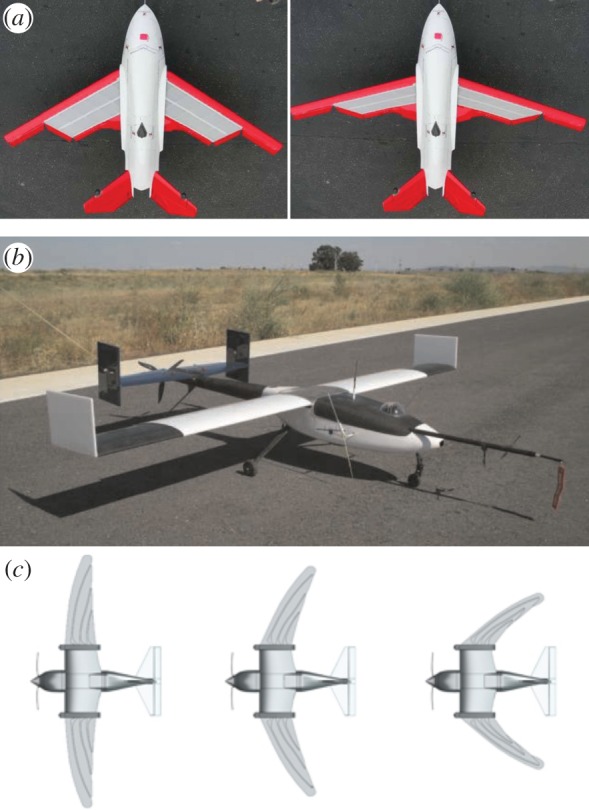
Morphing concepts: (*a*) the MFX-1 developed by NextGen [4], (*b*) VSW [5] and (*c*) RoboSwift [6].

The design of a morphing MAV requires numerous challenges to be addressed. A first challenge is to create a morphing surface that can undergo significant shape change without compromising the aerodynamic properties at the different operating conditions. A second challenge is that the mechanical constraints induced by wing morphing should not hinder platform control. Ailerons, for example, cannot be easily installed on variable-span wings and thus demand alternative solutions for roll control. A third challenge is that the design and manufacturing complexity of morphing mechanisms make it extremely difficult to find the right balance between aerodynamic efficiency and weight overhead. Therefore, despite extensive research in morphing technologies, only a few concepts have been experimentally assessed and only a small fraction have been successfully tested in flight [3]. The surface morphing vehicles that have reached sufficient maturity for flight tests fall in two design approaches: a continuous elastic skin supported by a mechanical structure and a rigid skin composed of several discrete elements. An example of the continuous elastic approach is given by the *Morphing Flight-vehicle Experimental* (*MFX-1*) developed by *NextGen* [4] (figure 1*a*): it features a scissor mechanism that affects span and sweep. It can achieve a maximum area change of 40% in 15 s, which is very slow for effectively changing the flight dynamics of a drone in cluttered environments. There are several examples of the discrete compositional approach, such as a telescopic wing whose surface can change up to 100% [7]. However, the pneumatic system used there is hardly scalable to an MAV. Another example, the *variable-span wing* (VSW) shown in figure 1*b* [5], uses two servomotors actuating an aluminium rack and pinion system that drives the extension/retraction of the outer wing. Despite a significant extension of the flight envelope, the slow dynamics of the sliding mechanism hinder the manoeuvrability of the aerial vehicle. Another example is given by *RoboSwift*, a morphing wing based on discrete feather-like elements inspired by swift birds [6] (figure 1*c*), which is able to fold its feathers backwards, thereby changing its wing area, sweep, slenderness and camber. However, to the best of the authors' knowledge, there are no data in the literature regarding the influence of this type of wing morphing on aerodynamic properties.

**Figure 2.**
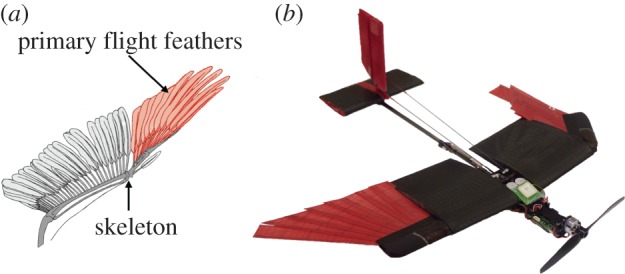
(*a*) Bird wings are composed of flight feathers connected to an articulated skeleton. The outermost feathers, known as primary flight feathers, significantly reduce the surface of the wing when folded [9]. (*b*) Prototype of the morphing wing drone described in this paper. Similar to birds, the drone is equipped with a feathered wing that folds the outermost sections in order to modify the surface area and also control roll angle for turning.

Several flying animals use morphing wings to improve flight capabilities. For example, birds exploit surface morphing to actively control their attitude and to achieve high aerodynamic performance within a wide range of flying speeds [8]. A bird wing is composed of an articulated skeleton controlled by muscles and covered with feathers that can overlap. The folding of the outermost feathers (primary flight feathers in figure 2*a*) enables a significant reduction in wing surface [9]. Foldable wings are found in birds with a mass spanning four orders of magnitude, from the *Ardeotis kori* weighing more than 10 kg (13.5–19 kg [10]) to the *Mellisuga helenae* weighing approximately 2 g [10].

In this paper, we describe a novel wing morphing mechanism inspired by the folding mechanism of bird feathers (figure 2). Similar to birds, the outermost part of the wing is equipped with artificial feathers that can be folded to actively change the surface of the wing. We show that this morphing mechanism can not only improve aerodynamic performance for manoeuvrability and wind resistance (§2), but also provide roll control with asymmetric folding of the two wings (figure 2*b*). In §3, we introduce the mechanical design of the proposed bioinspired wing and its integration in a small drone. In §4, we describe the aerodynamic design of the wing using a novel bird-like aerofoil. Computational simulations show the benefits of surface morphing for high-speed flight and manoeuvrability. In agreement with the computational results, wind tunnel characterization of a foldable wing prototype shows high lifting capabilities when fully deployed and a drag reduction up to 48% when the wing is fully folded. In §5, we show the effectiveness of asymmetric surface morphing (figure 2*b*) for controlling the roll dynamic of a morphing wing prototype. Asymmetric surface morphing has been compared to conventional ailerons using a computational model. In agreement with computational results, wind tunnel tests show that asymmetric folding is comparable to conventional ailerons for roll control at the low-speed flying condition. Finally, as a proof of concept, we validate the roll control authority of the proposed design with outdoor flights of a small drone with morphing wings.

## 2. Wing morphing to enhance manoeuvrability and wind resistance

Here, we discuss how an active change in the wing surface allows the coexistence of very different aerodynamic requirements, such as high manoeuvrability for flight among obstacles and high wind resistance for constant ground speed against strong headwinds.

**Figure 3.**
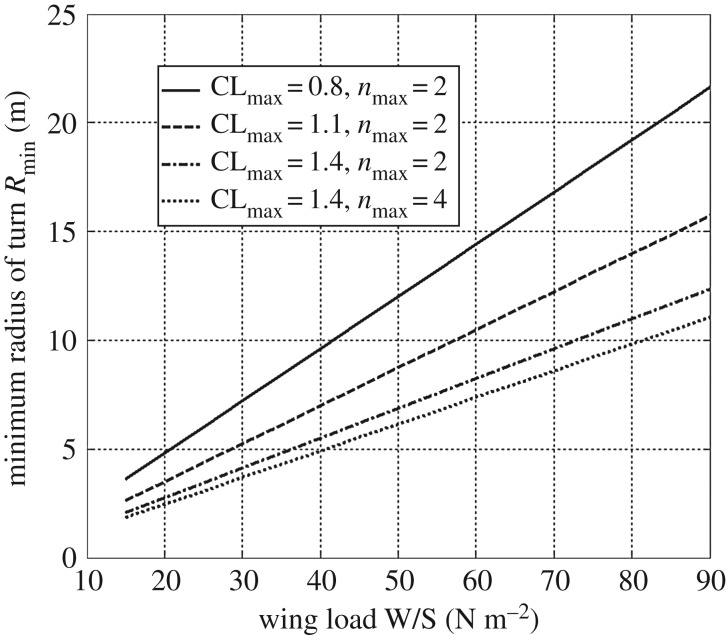
Minimum radius of turn *R*_min_ as a function of *n*_max_, CL_max_ and *W*/*S*.

Highly manoeuvrable MAVs can rapidly change course using a small turn radius. The describing equations of a turning manoeuvre can be found in [2]. For a coordinated horizontal turn, the minimum radius of turn can be defined as:2.1
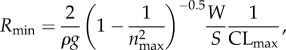
where *ρ* is the air density, *g* is the gravitational acceleration, CL_max_ is the maximum lift coefficient, *n*_max_ is the maximum structural load factor and *W/S* is the wing load (ratio of vehicle weight, *W*, to wing surface, *S*). The maximum load factor represents the ratio between the maximum lift the MAV structure can bear, divided by its weight, *W*. Based on equation (2.1), figure 3 shows the effect of CL_max_, *n*_max_ and wing load *W/S* on the minimum radius of turn. There are three ways to reduce turn radius and make an aircraft more manoeuvrable: high CL_max_, high structural load factor *n*_max_ and low *W*/*S*.

The maximum load factor, *n*_max_, has a lesser impact than the other two factors and increasing it would entail higher structural mass for the MAV. The second possibility is to increase the wing maximum lift coefficient CL_max_. It is worth noticing that high manoeuvrability is reached at a low-speed, low Reynolds condition for MAV category. In this flow regime, as detailed in [11] the maximum CL for an aerofoil with flap at *Re* = 10^5^ is only 1.5. Bioinspired feathered elements have been proposed as passive high lift devices in [12] but despite great potential, this technology needs further investigation. While a different approach proposed in [13] significantly increases the CL_max_, but shows considerable added weight to the system due to the need for a rolling shutter mechanism. The third possibility is to have a low wing load, *W/S*. For a given mass, a greater wing surface is required.

However, a greater wing surface requires more power to fly in the high-speed regime of the flight envelope. This would potentially affect wind resistance, defined as the capability to withstand both wind gusts and wind speed. Wind gusts affect MAV flight stability, increasing the probability of collision in cluttered environments. Similarly, even moderate breezes can affect the flight path and high-speed flight capability is beneficial to keep a constant forward ground speed in comparatively stronger headwinds. Wings with a small surface generate less frictional drag allowing an aerial vehicle to fly faster. A wing with a morphing surface could adapt its aerial surface to optimize aerodynamic performance to specific flight situations.

## 3. Mechanical design of foldable feathered wings and drone integration

Here, we propose a novel design based on foldable feathered wings in order to extend the flight envelope and control the roll angle of a drone. We start by describing the mechanical design of the wing folding mechanism and its integration into a drone.

**Figure 4.**
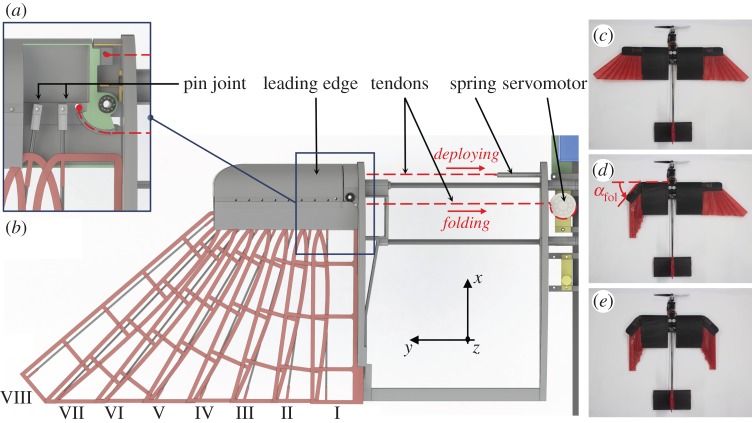
Mechanical design of the morphing wing. (*a*) Three-dimensional model of the left side of the wing with the main component involved in the actuation of the morphing section. For the sake of clarity, the Icarex™ cover on the feathers and the proximal section is not shown. (*b*) Local section of the leading edge to highlight the pin joints that link the feather to the thick leading section of the wing. Three extreme configurations of the wing: (*c*) fully extended, (*d*) asymmetric and (*e*) fully folded.

The mechanical design of the morphing wing is illustrated in figure 4. Each side of the wing is composed of an innermost fixed section and a feathered outermost section that can be actively folded. The feathered section is composed of eight artificial feathers connected to a leading edge. The feathered section can be actively folded by rotating the leading edge (figure 4*c*,*e*) with respect to the innermost fixed section (angle *α*_fol_ in figure 4*d*). The rotation of the leading edge is controlled by two tendons, shown as dashed lines in figure 4*a*, one to fold and the other to deploy the wing. The tendon for folding is directly driven by a servomotor (1810MG Digital Servomotor from HuiDa RC International Inc.), while the one for deployment is pulled by a pre-stretched linear spring. The pre-stretched spring limits the backlash of the mechanism allowing an angular accuracy between 0.3° and 0.5° to be achieved. The level of pre-stretch of the spring (3 N) counterbalances up to 1.5 times the drag force generated on the foldable section of the wing while flying at 20 m s^−1^. This level of pre-stretch is sufficient to avoid undesired yielding of the wing during flight with a safety factor of 1.5. The artificial feathers are connected to the leading edge through pin joints, except for the outermost feather (no. VIII) that is fixed. The pin joints allow the rotation of the feather during folding and their alignment with the feather no. VIII when the wing is completely closed (figure 4*e*). The feathers are composed of a straight carbon fibre shaft (diameter 1.5 mm) bonded to a fibreglass frame (FR4, thickness 0.2 mm) covered by a layer of Icarex™, which is a light and airtight ripstop polyester fabric. The same material also covers the fixed section of the wing. This design achieves a 41% surface reduction when completely folded (figure 4*e*).

The wing is integrated into a drone equipped with a frontal motor for propulsion (figure 2*b*). Roll is controlled by the asymmetric morphing of the wing (figure 4*d*) and pitch through a servomotor that moves an elevator located in the tail. The tail's vertical stabilizer is passive and ensures stability around the yaw axis. The drone is remotely controlled and is equipped with an electronic board that records motor and servomotors commands, attitude and GPS location for experimental measures presented in §5.3. The main characteristics of the drone are summarized in table 1.

**Table 1.** Main characteristics of the morphing MAV.

morphing MAV	fully extended	fully folded
weight (g)	330	
wing surface (m^2^)	0.131	0.077
wing span (m)	0.84	0.395
wing load (N m^−2^)	24.7	42.0
wing aspect ratio	5.4	2

The main design parameter for the morphing wing planform is the aspect ratio, which is the ratio between wing surface and the square of the wing span [2]. Aerofoil aerodynamic performance in terms of CL_max_ and efficiency degrade very rapidly below *Re* = 7 × 10^4^ [11], which was therefore selected as the inferior limit for the current design. For a prototype with the characteristic mass and wing load stated in table 1, an average wing chord of 0.16 m for the fully open configuration is necessary to limit the minimum *Re* number, resulting in an aspect ratio of 5.4 in the deployed configuration.

## 4. Drag and turn radius reduction

The effect of morphing on turn radius and wing drag obtained in simulations is discussed in this section along with the results from wind tunnel experiments on the morphing wing prototype.

**Figure 5.**
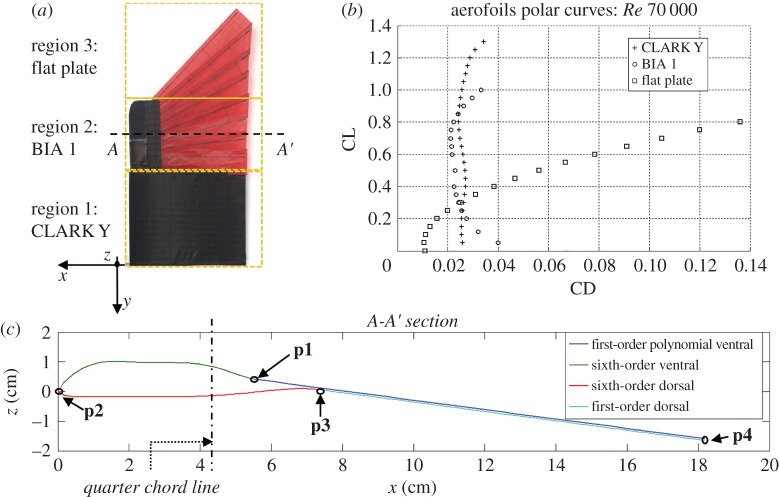
(*a*) The three regions with different aerofoil profiles over the wing. (*b*) Polar curves of the three aerofoils simulated with AVL at *Re* = 7 × 10^4^ which comprise the wing. (*c*) The novel BIA 1 corresponds to section *A*–*A*′. The different polynomials used to represent the aerofoil contour are shown in blue, green, red and light blue. The *x*-axis represents the distance from the leading edge in cm. **p1**, **p2** (leading edge), **p3** and **p4** (trailing edge) are the conjunction points between the polynomial curves used to model the aerofoil (see appendix A).

The wing is composed of three regions with different aerofoils (figure 5*a*). In §4.1, the lift–drag curves for the three aerofoils (figure 5*b*) are simulated using XFOIL, a computational tool widely used in aircraft design [14]. Then, as described in §4.2, the platform wing shape in the *x*–*y* plane (figure 5*a*) is simulated with a vortex lattice model (AVL, [15]) over a wide range of angles of attack. Combining the span-wise lift distribution obtained with AVL for the different angles of attack, with the aerofoils lift–drag curves, it is possible to obtain the overall drag of the wing as a function of lift (wing polar curve). The data of the wing polar curves allow the effect of morphing over turn radius and wing drag to be quantified. These data are compared with the results of wind tunnel experiments on the morphing wing prototype (§4.3).

### 4.1. Aerofoil aerodynamics design and simulations

As shown in figure 5*a*, the wing is composed of three regions with different aerofoils: region 1 corresponds to the non-foldable section of the wing, while regions 2 and 3 correspond to the foldable section of the wing. In the expected operating range of the morphing drone prototype, the corresponding minimum Reynolds number is *Re* = 7× 10^4^, which is also the minimum value used in simulations.

Region 1 is designed with a CLARK Y aerofoil [16]. At the low *Re* numbers of interest for the current design, this standard and well-known section shows good aerodynamic properties (high maximum lift coefficient and extended low-drag region, figure 5*b*).

Region 2 is composed of a thick leading edge (black area in figure 5*a*) that transitions into the thin and feathered trailing edge (red area in figure 5*a*) similar to the hand section of a bird wing. Aerofoil cross section is shown in figure 5*c*. Aerodynamic and geometric data on bird-like aerofoils are extremely scarce in the literature [17] and none of those found was considered suitable for the current design because biological aerofoils are cambered while the artificial feathers have a straight shaft for ease of manufacturing. Therefore, a novel bird inspired aerofoil (BIA 1) for the feathered section of the wing was developed (the aerodynamic design is detailed in appendix A). Key to the aerodynamic performance of the aerofoil is the thickness of the leading edge, around the quarter chord line and towards the leading edge. This thickness prevents flow separation in a wide range of angles of attack as shown by the corresponding polar curve represented in figure 5*b*. In comparison, a thin flat plate aerofoil would have led to poor aerodynamic performance except for a very limited range of angles of attack.

Region 3 of the morphing wing is a thin flat plate because of the lack of a thick frontal leading edge and the use of straight feather shafts. The polar curve for a flat plate is shown in square marks in figure 5*b*. As expected, the flat plate in the external region of the wing has higher drag and lower maximum lift coefficient than the bird-like aerofoil.

Both regions 2 and 3 are modelled as a flat plate when the wing is fully folded.

### 4.2. Aerodynamic simulations of the morphing wing

**Figure 6.**
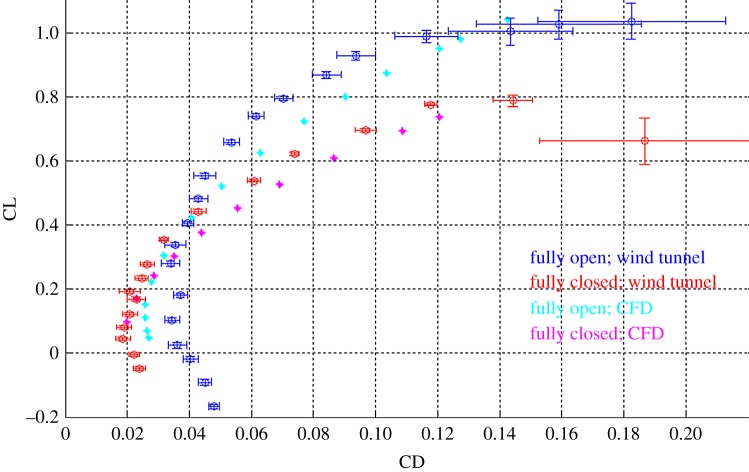
Polar curves for the morphing wing in the fully open and fully folded configurations (*Re* 70 000). The computational curves (cyan and magenta) has been obtained combining the lift distribution obtained in AVL simulations with the polar curves for the two-dimensional aerofoils computed with XFOIL (the morphing wing fully open is used as reference surface). Polar curves of the morphing wing at *Re* 70 000 obtained through wind tunnel measurements (blue and red).

The full wing has been simulated in two different configurations at maximum (fully open) and at minimum (fully closed) surface. The computational results are shown in figure 6 (cyan and magenta curves). For a direct comparison of the different configurations, the drag (CD) and lift (CL) coefficients are computed considering the surface of the fully open configuration as a reference. In the deployed configuration (figure 4*c*), the wing maximizes its surface and lift coefficient (CL) to achieve high manoeuvrability at low speed [2]. As a direct consequence, an aerial vehicle integrating the fully open wing would have a lower turn radius and therefore better manoeuvrability than one with the fully folded wing. Introducing the CL_max_ obtained in simulations for the two configurations in equation (2.1) §2, the prototype implementing the morphing wing has a minimum turn radius of 3.9 m with the wing fully extended and 6.6 m when fully folded (load factor of 3, §2). Also using the CL_max_ obtained in simulations, the prototype would have a minimum speed of 6.3 m s^−1^ with the wing fully extended and 7.6 m s^−1^ when the wing is fully folded.

In the folded configuration (figure 3*e*), the wing minimizes its surface and drag coefficient (CD) to allow high-speed flight. Folding the wing is beneficial at low CL values, where the CD_min_ (0.021) is reduced by 29.3% with respect to the corresponding value in the deployed configuration (0.027). In this regime, the major drag component is parasitic drag. A CD reduction would enable faster flight.

### 4.3. Wind tunnel tests of drag reduction and comparison with simulations

Wind tunnel tests of the morphing wing have been carried out in the HEPIA wind tunnel in Geneva. The morphing wing has been tested at three Reynolds numbers (70 000, 121 000 and 175 000) within the expected operational range for the developed prototype. Wings were mounted onto a custom-made sting balance and placed in the HEPIA wind tunnel, which has an octagonal test section of 2.0 × 1.5 m. The balance is a strain-gauge three-component balance, which provides values for lift, drag and roll torque. The balance was designed specifically with full scale corresponding to the maximum range of force and torque values expected in the experiments (max. measured force and torque, 13.7 N and 1.4 Nm). The tests were run at air speeds between 6.9 and 17 m s^−1^ (corresponding to a Reynolds number of 70 000–175 000). Each force/torque value was sampled at 300 Hz for 8 s at each angle of attack *α* (0° → 30°, −6° → 0°, Δ*α* = 1.5°, precision less than 0.5°). We verified that no hysteresis phenomenon was present during testing, thus obtaining very good repeatability of measurements.

The polar curves for fully open (blue) and fully folded (red) wing configurations at Reynolds numbers of 70 000 are shown in figure 6. Lift and drag coefficients take as a reference the surface of the fully open wing. As expected, the fully open configuration produces higher lift than the fully folded configuration as underlined by the higher CL_max_. Furthermore, the fully closed wing shows a lower drag coefficient CD_min_ in the low CL region (left area of the polar curve).

The maximum CL values measured experimentally are similar to those found in simulations. For drag, however, some discrepancies appear. The measured reduction in CD_min_ associated with wing folding (45.1%) is higher than the one obtained through computational modelling (29.3%). In fact, while the measured CD_min_ for the fully closed configuration (0.020 ± 0.001) is well in agreement with the computational results (0.021), the measured CD_min_ for the fully open configuration (0.033 ± 0.004) is higher than the computed values (0.027). The computational model underestimates the drag of the fully open wing in the low CL condition. As discussed in [18], a possible explanation is that the artificial feather-like elements overlap with each other but do not adhere, unlike natural feathers. Therefore, the incoming air can flow in-between the overlapping portion of the feathers increasing the frictional drag due to an increase in the effective surface. For the fully open wing, interlocking feathers could potentially reduce frictional drag also at high CL. However, its effect on pressure drag is not clear and a definitive answer would need further investigations. The inaccuracy related to the manufacturing process of the wing, especially for the hand part could also be responsible for the observed discrepancy.

In the high CL region, the tested open configuration shows lower drag than the computational model. Isolated testing of the BIA 1 aerofoil and a comparison with XFOIL results would help elucidate the cause of the discrepancy. Experimental and computational drag values for the fully folded configuration are in good agreement with a slight tendency of the model to overestimate the drag at medium and high CL. The use of more powerful computational methods for a more accurate aerodynamic wing modelling is still a challenge due to the transitional *Re* range experienced by the wing. However, the fully closed configuration is expected to operate at low CL, where the computational model results are in close agreement with experimental measurements.

Polar curves for Reynolds numbers of 121 000 and 175 000 (appendix B) are qualitatively very similar to figure 6 while the main quantitative differences are summarized in table 2. The reduction in CD_min_ due to wing folding is almost constant with *Re*. The wing maximum lift coefficient CL_max_ increases with *Re* number in agreement with results in the literature [11]. For safety reasons, the CL_max_ at the highest *Re* number was not tested to avoid wing failure. The lack of data in this region (high CL, high *Re* numbers) is not a problem because it is not expected to be within the operating range of a possible morphing vehicle.

**Table 2.** The effect of Reynolds number on the morphing wing's polar curves.

Reynolds number	70 000	121 000	175 000
CD_min_ reduction (%)	−45.1	−48.2	−47.5
CL_max_ (fully open)	1.01	1.06	n.a.

## 5. Roll control

Although it is difficult to install conventional ailerons on the morphing wing described here, we show that asymmetric folding of the two wings can be used to effectively control the roll angle of the drone (figure 4*e*). Here, we compare this strategy to conventional ailerons based on the roll torque coefficient and roll dynamics in simulation and wind tunnel tests; finally, we validate the use of asymmetric folding for roll control with outdoor flight tests of the drone.

### 5.1. Computational model

**Figure 7.**
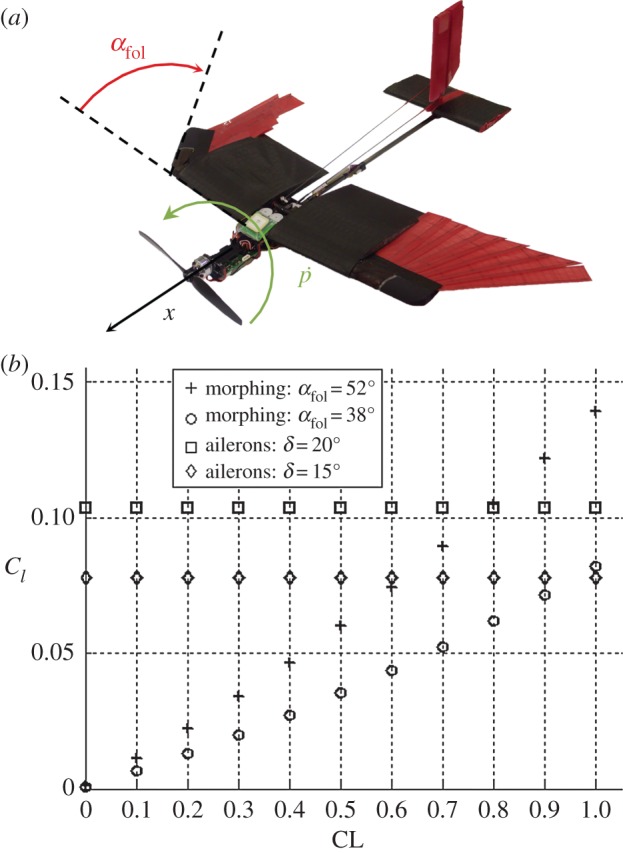
(*a*) Asymmetric surface morphing to generate a rolling torque. (*b*) Roll torque coefficient *C_l_*: comparison between ailerons and asymmetric surface morphing simulated with AVL (*C*_*l* ail_/*∂δ* = 0.297 rad^−1^).

A pure roll manoeuvre can be described through the first-order differential equation as in [19]:5.1
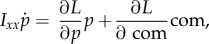
where *I_xx_* is the mass moment of inertia of the vehicle around the roll axis (*x*-axis), 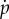 is the roll acceleration and *L* the roll torque. The inertial damping/excitation term due to coupling between the roll rate and inertia change has been neglected. This term, which is present especially for the morphing case, is more than one order of magnitude smaller than the aerodynamic damping [20]. On the right-hand side of the equation, the term 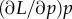 represents the aerodynamic damping moment [20]. The term 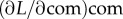 is the rolling moment due to a roll command com representing either aileron deflection, *δ*, or folding angle, *α*_fol_ of the feathered wing (figure 7*a*) depending on the roll control mechanism considered. For both mechanisms,5.2
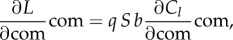
where the roll torque coefficient *C_l_* is *C_l_*_morph_ or *C*_*l* ail_ depending on the roll control mechanism. The folding angle *α*_fol_ is represented in figure 7*a* defined as the angle between the leading edges of the fixed and moving sections of a semi-wing. The terms *b*, *S* and *q* are the reference wing span, wing surface and dynamic pressure, respectively.

In the following analysis, the roll control ability of asymmetric morphing is compared to a reference conventional wing with ailerons and no morphing capabilities. The reference wing has the same planform geometry as the morphing wing in the maximum surface configuration. Aileron sizing has been performed in order to give very high roll control authority [21] extending from the trailing edge up to 25% of the wing chord and along 65% of the overall wing span.

The roll torque coefficients *C*_*l* ail_ and *C*_*l* morph_ have been simulated with AVL. For asymmetric morphing, the left semi-wing is fully extended while the right is folded at *α*_fol_. Simulation results presented in figure 7*b* underline the difference between asymmetric morphing and ailerons for roll torque generation: *C*_*l* ail_ is independent of CL (in the range of CL tested) and is only affected by the deflection angle *δ* while *C*_*l* morph_ depends not only on the folding angle *α*_fol_ but also on CL.

As previously discussed, high manoeuvrability is achieved flying at high CL, which corresponds to the right side of figure 7*b.* This is also where asymmetric surface morphing is able to generate roll torque coefficients comparable or superior to ailerons. On the downside, in the far left region of the graph corresponding to low CL, ailerons generate much higher *C_l_* than asymmetric morphing. Therefore, ailerons are more effective for roll control than asymmetric morphing when flying at high speed (low CL region).

A model for roll dynamics has been implemented in MATLAB Simulink^®^ in order to compare the roll rate and bank angles obtained while performing a pure roll manoeuvre with the two mechanisms. The computational model was created for several reasons. First, ailerons and asymmetric morphing have different time responses and the model shows the impact of this factor over roll dynamics. Moreover, the morphing prototype is remotely piloted and therefore, it is not possible to obtain a horizontal roll manoeuvre. In this case, modelling the roll dynamics was the only solution to have a quantitative comparison between the two roll mechanisms for the dynamics of the bank angle.

One of the main factors influencing the ability to effectively control roll dynamics through asymmetric morphing is the time required for wing morphing. In fact, after the command to turn has been given to the servomotors, the lower the time required for folding, the lower the distance travelled by the aerial vehicle before turning and therefore the space required for the manoeuvre.

The time required to actuate the morphing wing used in this analysis has been measured in ground testing experiments as reported in table 3. The opening and closing times of the morphing wing were measured under various load factors. The load applied to the aerodynamic force centre of the external morphing part of the wing is based on load factor and the weight of the morphing drone prototype (table 1). The difference in the opening and closing time is due to the difference in actuation (folding is driven by a servomotor, deploying by the spring). As expected, increasing the load factor increases friction in the mechanism, thus slowing down the actuation times. Concerning the conventional wing with ailerons and no morphing capabilities, the time required to actuate the aileron deflection is taken into account using a ramp with the slope (1°/0.05 s) observed in a vehicle with characteristics similar to the prototype (eBee^™^ by senseFly [22]).

**Table 3.** Full stroke actuation times (and standard deviation over four measurements) of the morphing wing using 1810MG Digital Servo from HuiDa RC International Inc. The time constant of the step response is between 110 and 140 ms, respectively, for low and high load factors.

	load factor (g)				
	0	1	2	3	4
opening time *T*_o_ (ms)	136.6 ± 26.2	146.6 ± 4.7	180 ± 35.6	200 ± 1.2	206.6 ± 36.8
closing time *T*_c_ (ms)	156.6 ± 4.7	176.6 ± 4.7	186.6 ± 18.8	183.3 ± 23.5	186.6 ± 9.4

To perform the dynamic analysis, the aerodynamic damping moment 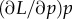 has been computed using AVL for both the morphing wing and the reference wing and the roll inertia is the same as for the prototype presented in figure 2*b.* Furthermore, as for a given vehicle, manoeuvrability is maximized while flying at the maximum lift coefficient (see §2), the roll torque coefficient produced by asymmetric morphing corresponds to this condition in the current analysis. Based on figure 7*b*, the values are *C*_*l* ail_ (*δ* = 20°) = 0.104 and *C*_*l* morph_ (*α*_fol_ = 52°) = 0.139.

**Figure 8.**
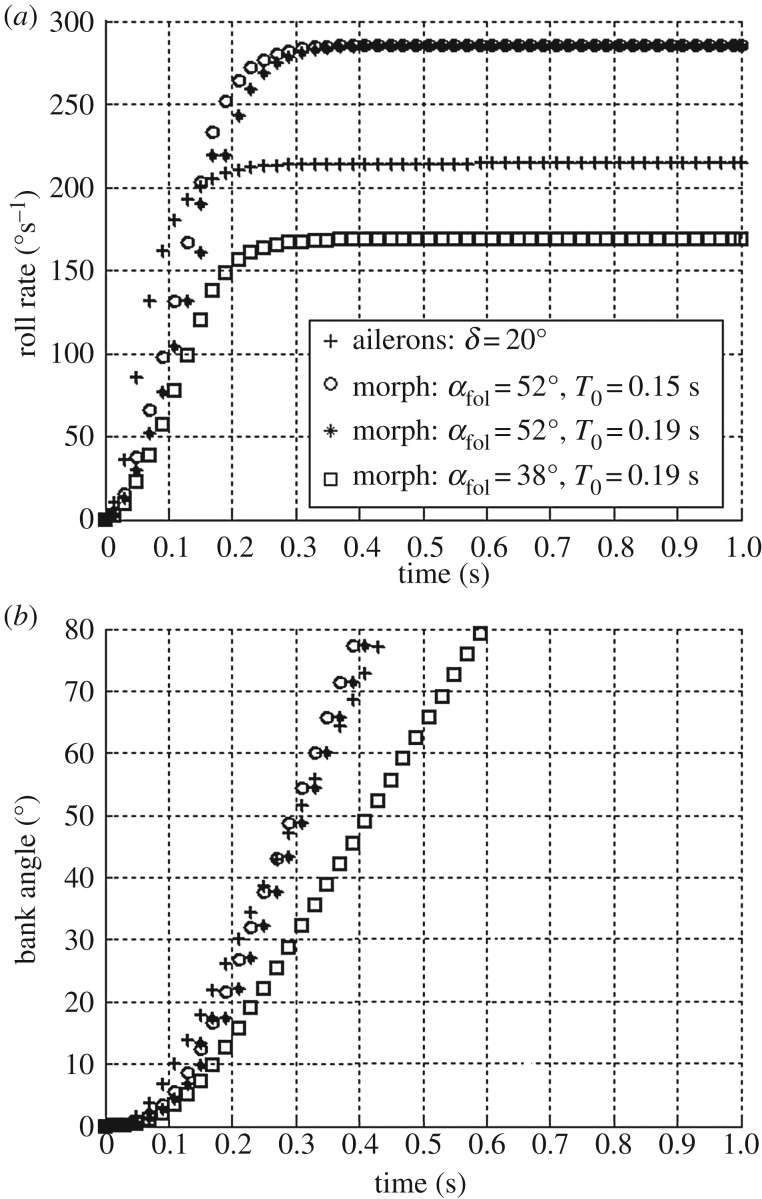
Maximum roll rate and bank angle comparison: ailerons (*δ* = 20°) and asymmetric morphing (*α*_fol_ = 52°) for *T*_o_ corresponding to a load factor of 0 and 4. The value *I_xx_* = 0.010 kg m^–2^ is used in the simulations which corresponds to the inertia of the morphing prototype with fully open wing.

Figure 8 shows the roll rate and bank angle evolutions for ailerons and asymmetric morphing. Despite a slightly slower actuation time, wing morphing can generate maximum roll rates, which are higher than conventional ailerons (figure 8*a*). Increased actuation time due to higher load factors is a less critical factor than the folding angle for the bank angle dynamics as also shown in figure 8*a*. This holds true because, irrespective of the tested load factor, the actuation time of the proposed morphing mechanism is always comparable to the one of conventional ailerons.

Figure 8*b* shows that for wide turns requiring small bank angles, both mechanisms have similar time requirements for banking, while for very sharp turns, and therefore high banking angles, asymmetric morphing is faster than ailerons. For example, a vehicle using ailerons requires 0.17 s more to reach a bank angle of 75° (load factor of 3) and therefore travels for about 0.5 m more before reaching the smallest turn radius compared to morphing (flying at CL_max_).

In summary, when flying at high CL, in the conditions of maximum manoeuvrability, asymmetric surface morphing is comparable or better than conventional ailerons for roll control as also shown by the bank angle dynamics in figure 8*b*. On the downside, as the roll torque coefficient heads towards 0 for very small CL (figure 7*b*), asymmetric morphing alone is not effective to control roll dynamics in this flying condition. A possible solution is to couple asymmetric morphing with aircraft pitch-up in order to increase the instantaneous CL and consequently the roll torque coefficient.

### 5.2. Wind tunnel tests

**Figure 9.**
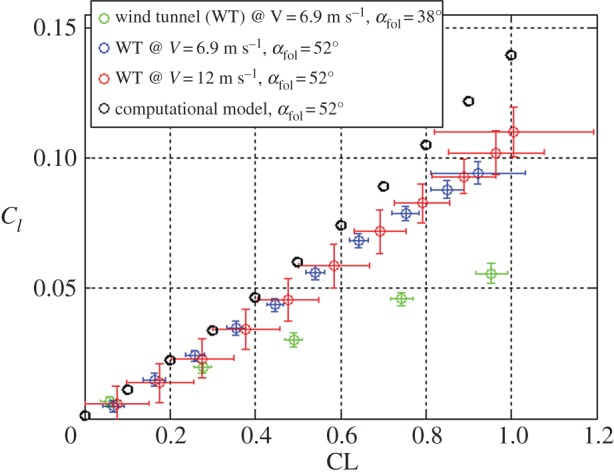
Roll torque coefficient as a function of CL: one semi-wing fully open while the other is folded at an angle *α*_fol_ = 52° for two different speeds (blue, red) and 38° (green). The results of the computational model for *α*_fol_ = 52° are in black.

The roll torque generated by asymmetric morphing has been measured experimentally in the wind tunnel: one semi-wing was fully open while the other was folded at two different angles *α*_fol_ (38° and 52°). Figure 9 shows *C*_*l* morph_ as a function of wing lift coefficient CL measured for different *α*_fol_ and wind speeds. Also shown are the results from the computational model. As already discussed in §5.1, the roll torque coefficient always increases with lift coefficient. The measured values for *C*_*l* morph_ (*α*_fol_ = 52°, wind speed 6.9 m s^−1^) are lower than expected from the computational model and the discrepancy increases with CL. This could be due to structural flexibility of the wing or to an early stall over the external part of the open semi-wing. Structural flexibility can cause the wing to twist, which results in a reduction of the effective angle of attack and of the roll torque coefficient. The effect of twisting is amplified at high speed where the wing is subjected to higher aerodynamic loads. However, the experimental data do not show a significant reduction of *C*_*l* morph_ (*α*_fol_ = 52°) when transitioning from 6.9 to 12 m s^−1^. Therefore, early stall in the external part of the wing (regions 2 and 3, figure 5*a*) more than wing flexibility is very likely to be the main reason for the discrepancy between computational and experimental results. In future design, the problem of early stall can be addressed by using a cambered shaft for the implementation of the artificial feathers.

### 5.3. Flight tests

**Figure 10.**
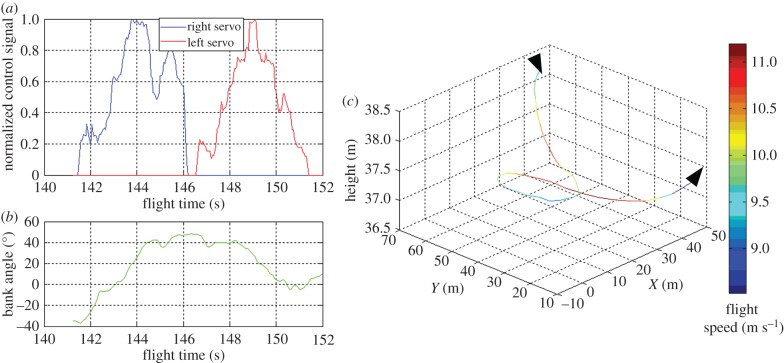
(*a*) Servomotor control signal, (*b*) bank angle and (*c*) flight trajectory during a turn manoeuvre in the flight time section of 141–152 s. The data were recorded by an on-board autopilot equipped with IMU and GPS.

The morphing prototype underwent flight tests to demonstrate the ability to successfully control MAV roll dynamics using asymmetric span morphing and to perform bank turns. A flight of 6 min was performed, including an arm throw take-off and a ground landing. Attitude and flight trajectory of the MAV, and servomotor control signals were recorded by an electronic board (http://lis-epfl.github.io/MAVRIC_Library/) hosted in the MAV and equipped with IMU and GPS. Figure 10 depicts a roll manoeuvre performed in the time range between 146 and 152 s measured from takeoff. Figure 10*a* shows the time history of commands for the servomotors controlling the folding of the left and right morphing mechanism*.* The external part of the semi-wing is fully opened or fully closed via a null or maximum command of the respective servomotor. The evolution of the bank angle over time is shown in figure 10*b*, and the trajectory of the prototype is shown in figure 10*c.* Asymmetric morphing allows control of the bank angle as shown in figure 10*a*: fully folding the right semi-wing causes an increase in the bank angle (142–144 s) while fully folding the left semi-wing causes a reduction (146–150 s). The resulting MAV turning manoeuvre is evident when looking at the trajectory shown in figure 10*c*. Moreover, the pilot verified the control effectiveness of the morphing surface at low speeds in agreement with the models derived from computations and wind tunnel testing. As expected, roll control effectiveness degrades at higher speeds (compared with traditional ailerons). In these conditions, however, the pilot was able to dramatically increase roll control performing a pitch up manoeuvre before rolling. This behaviour underlines the necessity to develop specific control laws in order to obtain the best from morphing technologies.

## 6. Conclusion

We have developed a morphing wing that can change wing surface, in order to improve low-speed manoeuvrability as well as enhance high-speed performance for wind rejection. The fulfilment of these opposing requirements has been made possible thanks to a feathered structure that can undergo a 41% reduction in the total wing surface when folded. In the fully deployed configuration, the wing has a large surface and 32% higher lift coefficient. On the other hand, when fully folded, the wing reduces the minimum drag coefficient of more than 40%. Drag reduction in the low CL region is expected to enhance the maximum speed of the aerial vehicle. Although conventional ailerons cannot be installed on the wing, the morphing mechanism can be actuated asymmetrically to provide roll control authority.

In the short term, several design modifications could improve the current design. For the fully open configuration, the use of curved feather shafts would allow an increase in CL_max_, and therefore manoeuvrability, while also increasing aerodynamic efficiency at high CL. The use of feathered elements for the leading edge part of the external wing, also observed in birds, would reduce the CD_min_ of the folded wing. Moreover, an ad-hoc autopilot must be developed to perform roll control with asymmetric morphing also considering the coupling with a pitch-up manoeuvre to increase control effectiveness in the low CL flying conditions.

In the long term, the full potential of morphing wings could take advantage of new materials, design strategies and control algorithms. For instance, the current wing relies on a traditional mechanical design with hinges and tendons, which is intrinsically fragile, complex to manufacture and difficult to scale down. The use of innovative designs based on origami manufacturing or variable stiffness materials could provide significant benefits towards more robust and integrated morphing wings. In addition, specific control algorithms are required to take advantage of fast morphing for control authority and for autonomous adaptation to different environmental conditions.

## Supplementary Material

ESM_2_Picture.jpg

## Supplementary Material

ESM_3_Data_supporting_the_manuscript.m

## Supplementary Material

Response to Referees.pdf

## Figures and Tables

**Figure 11.**
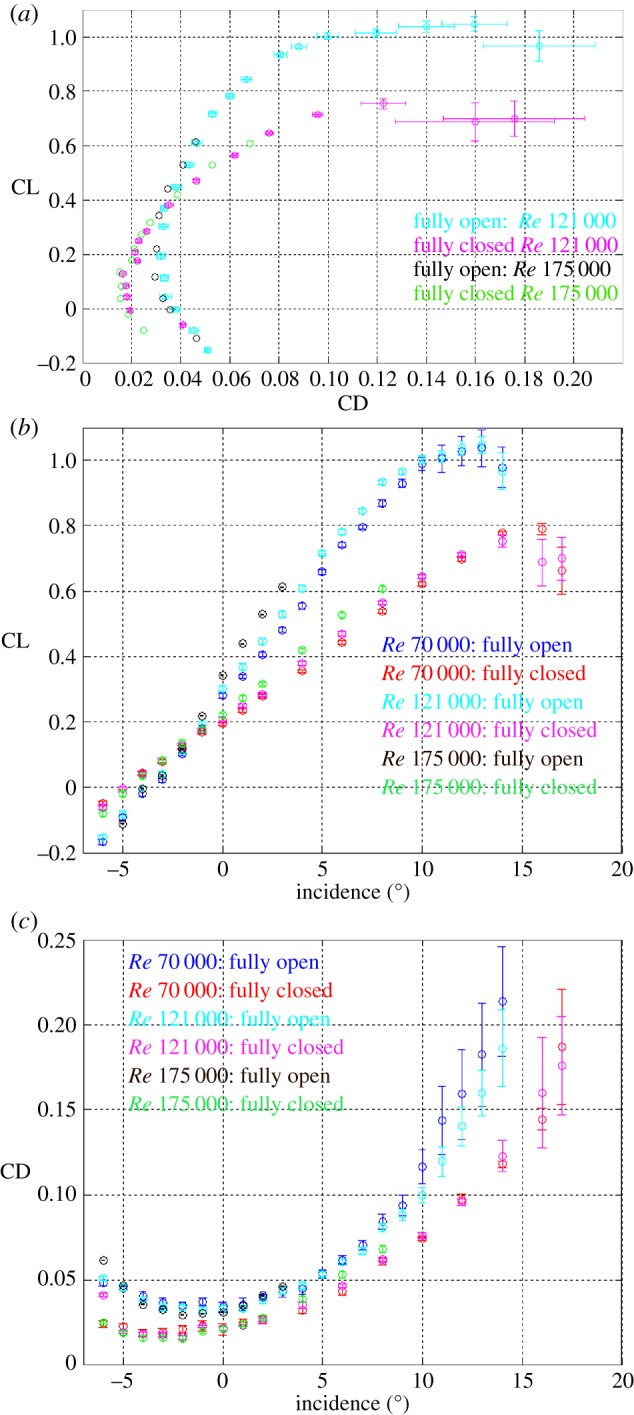
(*a*) Polar curves of the morphing wing obtained through wind tunnel measurements for *Re* 121 000 and 175 000 (error bars are fully contained inside the circular markers and therefore not shown). (*b*) CL and (*c*) CD as a function of incidence for the three tested values of Reynolds numbers (70 000, 121 000 and 175 000).

## Appendix A

The geometry of the BIA 1 aerofoil was derived from a design algorithm specifically developed in Matlab^®^. The main design steps are summarized below:

— The geometry of the aerofoil was parametrized using four polynomials (figure 5*c*). The dorsal and ventral thick portions of the leading edge are represented by two sixth-order polynomials (green and red lines in figure 5*c*) as detailed in [23]. The thin rear part of the aerofoil is modelled using two first-order polynomials (blue and light blue lines in figure 5*c*). To ensure surface continuity, the polynomials merge in p1, p2, p3 and p4. At p1, p2 and p3, the polynomials passing through these points have the same tangent. In total, the geometry was described using 11 parameters.
— The searching space for the parameters describing the thick part was obtained starting from the parameters describing the NACA 4410 geometry [23]. As explained in the following steps, the search space for the parameters was determined using a trial and error process.
— Aerofoil geometries were obtained from randomly selected parameters within the search space and simulated with XFOIL. The search space boundaries for the parameters were modified in order to obtain XFOIL convergence for more than 95% of the aerofoils.
— The final aerofoil BIA 1 (figure 5*c*) was selected based on the best power factor CL^3/2^/CD among 5000 aerofoils.

## Appendix B

The polar curves for fully open and fully folded wing configurations at Reynolds 121 000 and 175 000 are shown in figure 11*a*. Polar curves are qualitatively very similar to figure 6 (Reynolds 70 000) while the main quantitative differences are summarized in table 2. Figure 11*b*,*c* shows the lift and drag coefficients for the fully open and folded wing measured at *Re* 70 000, 121 000 and 175 000. For a direct comparison of the different configurations, the drag (CD) and lift (CL) coefficients are computed considering the surface of the fully open configuration as a reference.
