# Supplementary figures and images for: Bioinspired morphing wings for extended flight envelope and roll control of small drones
Source: Interface Focus. 2017 Feb 6;7(1):20160092. doi: 10.1098/rsfs.2016.0092 (PMC5206609; doi:10.1098/rsfs.2016.0092)

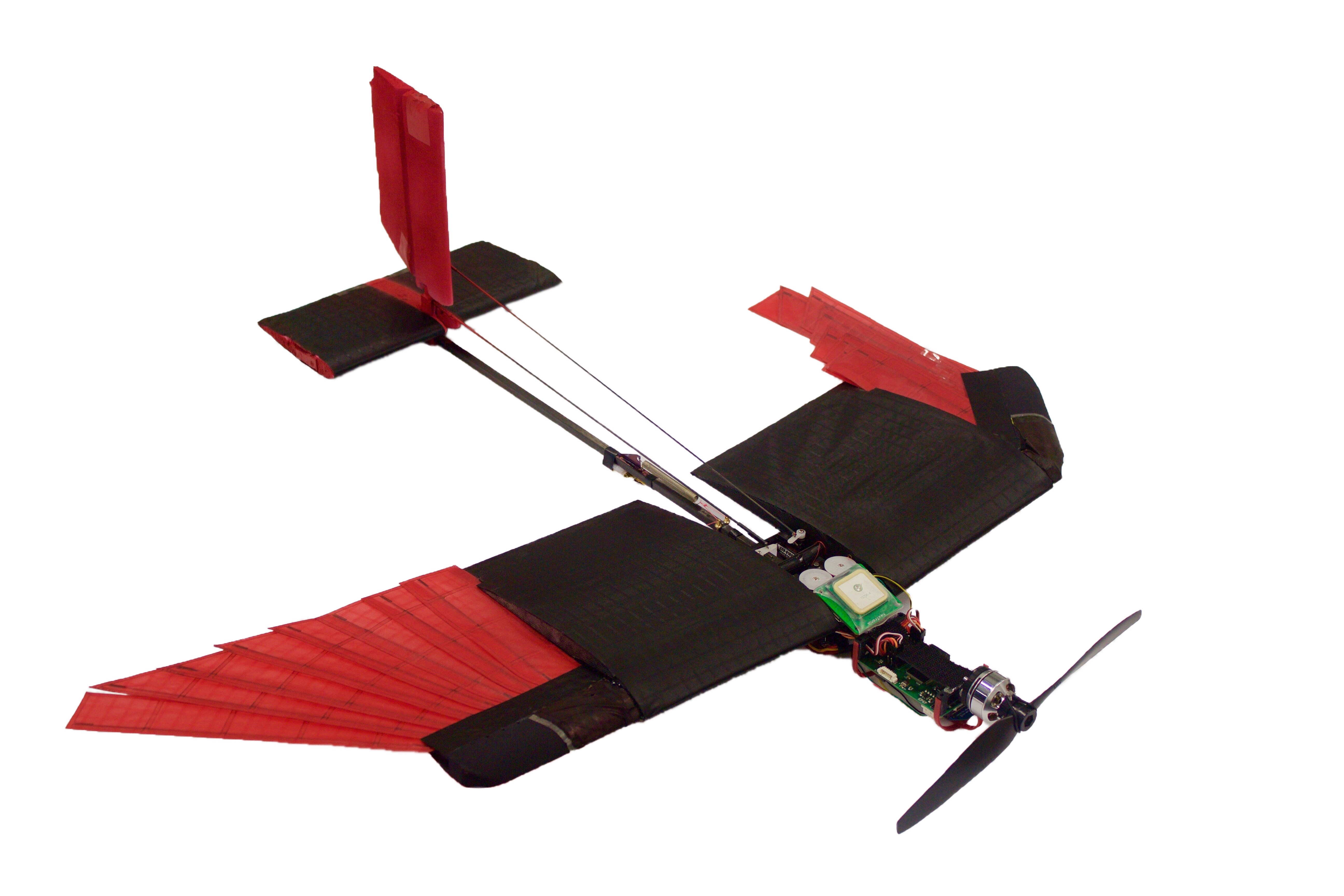

Supplement: ESM_2_Picture.jpg [file rsfs20160092supp1.jpg]
