# Supplementary material for: Bioinspired morphing wings for extended flight envelope and roll control of small drones
Source: Interface Focus. 2017 Feb 6;7(1):20160092. doi: 10.1098/rsfs.2016.0092 (PMC5206609; doi:10.1098/rsfs.2016.0092)
Supplement: Response to Referees.pdf [file rsfs20160092supp3.pdf]

Reviewer 1

We would like to show our appreciation to the reviewer for her/his useful feedback, which has allowed us to improve the manuscript.

We have replied to each request from the review below highlighting in blue our response.

The paper reports on the design and aerodynamic characteristics of a surface-morphing wing for aerial vehicles. A computational model for the morphing wing is proposed and tested against wind tunnel data. An analysis on roll control authority of fixed-wing aerial vehicles equipped with such morphing wings is also provided, while manual testing flight with a prototype vehicle shows that asymmetric wing morphing can lead to roll maneuvers in practice.

While wind tunnel testing confirms that the reported wing design reduces the minimum drag, the proposed model appears to need further refinements to improve its accuracy. At its current form, the simulated drag polars (Fig. 5a) differ both qualitatively and quantitatively from the experimental ones (Figs. 5b and 11). To better judge on the accuracy of the model it is important to plot the lift polars alongside drag polars. Since actual data from the wind tunnel testing are readily available, it is not clear why not to revise the approach outlined in Section 3-A and Appendix B to create a computational model that yields more accurate polars. The above discrepancy may also invalidate the comparisons on the roll control efficacy of asymmetric morphing and conventional ailerons.

We agree with the reviewer that the proposed computational model had to be further refined. We have updated the model: due to inaccurate interpolation of CLARK Y airfoil polar curve, wing drag at high CL was considerably overestimated for both open and folded configurations. Moreover, an error in the computation of the induced drag when importing data from the vortex lattice software was also found. This error resulted in underestimating the drag of the folded wing at medium and high CL values. Data closer to the stall region have also been added for the closed configuration.

Overall, the revised computational model shows better accuracy in matching wind tunnel measurements. As suggested by the reviewer, polar curves from experiments and computations are plotted alongside (Figure 6) for an easier direct comparison. Some discrepancies are still present: the revised model tends to overestimate wing drag at high values of CL for both configurations and underestimates minimum drag for the wing in the open configuration. The latter may also be due to inaccuracy in the manufacturing of the wing, especially the hand part which consists of several elements (feathers` shafts, joints and leading edge component) assembled together. More computationally expensive aerodynamic tools should be used to further improve the accuracy of the aerodynamic model.

In the current analysis, however, the efficacy of asymmetric morphing in roll control depends solely on the lift distribution over the wing (and its asymmetry). Input for the analysis is the roll torque coefficient and the computational model shows good agreement with experimental results (Figure 9 of the latest version of the paper). The comparisons on roll control efficacy between asymmetric morphing and conventional ailerons is therefore not affected by the existing drag discrepancies between the model and the measurements.

We discussed the possibility to use roll torque coefficients obtained from experiments in the efficacy analysis. For this reason, we also tested a wing with conventional ailerons (data not published). We found discrepancies between theoretical models and measurements due to wing flexibility. Since most small MAVs have relatively flexible wings (e.g. eBee™ by senseFly), at this stage, we decided to use computational data which do not account for flexibility in order to guarantee repeatability of the analysis.

Regarding the actual wing design, it would be useful to provide further details on the pre-stretched spring. How is the amount of pre-tension calculated, and how is it related to the expected operational speed of the vehicle? It is conceivable that the wing may passively yield when the robot is flying at high speeds if the amount of tension is not sufficient. But, conversely, increasing the amount of pre-tension hinders wing folding; the folding time should be increased, while it is possible that stronger servos may be needed (in turn affecting the vehicle's weight, and thus flight time and performance). This is an interesting design space that it could be explored in greater detail in this paper.

During the design of the folding mechanism, the authors have thoroughly evaluated three different transmission mechanisms: a pull/push rod, a bowden cable and a tendon driven mechanism coupled with a spring. The first two solutions provide a direct control on wings motion, but have their own drawbacks, such as backlash for the pull/push rod and high friction and tearing of the bowden cable. The tendon driven mechanism lack of a direct control of the folding movement that is passively driven by the spring. On the other hand, this solution is simple to implement, has, no backlash, limited friction and increases the resilience of the wing allowing a passive folding during collisions, for example during the landing. This feature is desirable especially in the development phase.

We have updated the text by explaining how the amount of pre-stretch of the spring is calculated. The level of pre-stretch (3 N) of the spring counterbalances up to 1.5 times the maximum folding moment (0.045 Nm) generated by the drag force on the foldable section of the wing while flying at 20 m/s. This level of pre-stretch is sufficient to avoid undesired yielding of the wing during flight with a safety factor of 1.5, while limiting the need for a strong and heavy servomotor.

Interestingly, in a push/pull rod configuration, the servomotor would have required the same amount of force to ensure the deployment of the wing in the worst case scenario with a 1.5 safety factor

Last but not least, Appendix C must be in the main text; at the current paper form, important information regarding Fig. 5 can only be found in the Appendix and not in the main text. Appendix A could also be moved into the main text.

We agree with the reviewer: appendix A has been moved in the text and the important information regarding Fig.5 have been moved from appendix C to the main text.

Minor comments:

- Please proofread the manuscript carefully (e.g., CL_max is used before being defined, the wing load is only defined in Appendix A, reference [16] uses brackets but all other references use parentheses, and some typos exist---for example "platform" right above Section 3-A, spacing in Fig. 4 captions, "shown" in Section 3-B).

The manuscript has been carefully checked, wing load and CL_max are defined when they are first used in the text. Typos have been corrected.

- Please revise some entries in the references to add missing pages, remove duplicate years, etc.

Entries in the references have been revised.

Reviewer 2
We would like to show our appreciation to the reviewer for her/his useful feedback, which has allowed us to improve the manuscript.
We have replied to each request from the review below highlighting in blue our response.

Page 3, line 27 : indicate model and company of the servomotor
We agree with the reviewer and the text has been updated with clear reference to model and manufacturer of the servomotor.

Page 3, line 29: indicate model and company of the pin joint
The pin joint is custom manufactured. It is composed of a carbon fiber pin obtained by a pultruded carbon rod with a diameter of 2 mm. The pin connects the leading edge with a 3D printed joint that hosts the shaft of the feather. The pin is free to rotate with respect to the leading edge and it is forced inside the 3D printed joint.

Section 2: please give the accuracy in the leading edge angular positioning. Is there any backlash?
The leading edge is actuated by the servomotor and a tendon driven transmission incorporating a pre-stretched spring. The intrinsic low backlash of the servomotor and the pre-stretched spring ensure a negligible backlash in the movement of the leading edge. As a consequence, the angular position accuracy of the leading edge depends on the angular resolution of the servomotor, which, according to the datasheet of the manufacturer is comprised between 0.3° and 0.5°.

Section 2: please give the dynamics of the leading edge control: time constant of the stp response, time to fully fold and unfold the wing.
The time to fully fold and unfold the wing is reported in Table 3. The folding and unfolding time have been measured from high speed video (240 fps) of the wing's motion. We have reanalyzed the videos and measured the time constant of the stp response considered as the time to reach 63% of the final value (fully folder or fully deployed wing). The time constant is comprised between 110 and 140 ms, respectively for low and high load factors. These data have been integrated in the text.

Page 6, line 9: flight speed range (simulation and real conditions) must be provided (idem for figure 9c).
The figure showing the turn trajectory (Figure 10c) has been updated adding the flight speed during the maneuver.
The prototype minimum flight speed is 6.1 and 6.3 m/s using respectively the maximum lift coefficient from simulation and wind tunnel testing of the wing. The manuscript has been updated to include this information.
Maximum speed, which is affected by drag and available thrust, has not been determined in simulation and flight tests. Drag is mainly due to the wing, the fuselage and control surfaces. In this study we focused on reducing the wing drag via surface morphing and polar curves from simulation and wind tunnel testing relates to the wing only.
The percentage increase in maximum speed (difference between maximum speed of the prototype with wing folded and open, divided by the maximum speed with wing open) depends upon the efficiency curve (efficiency vs flight speed) of the propulsion system. The propulsion system is composed by the electronic speed controller, the electric motor and the propeller. A detailed analysis with the selection of these components and their impact on the flight range will be the object of a further study also taking into account the fuselage design and control surfaces optimal design.

To be definitely convinced by the ability of the new folding mechanism to control the roll, I

would like to see the response of an IMU embedded onboard the drone during roll manoeuvres. Bank angle and control signals are not sufficient.
The bank angle shown in Fig. 10b is computed using the data gathered by an IMU embedded onboard of the drone. This has been clarified in the main text of the article. The drone is equipped with an autopilot developed internally in the lab (http://lis-epfl.github.io/MAVRIC_Library/), that records data from the IMU. The bank angle is obtained by fusing the data from a 3 axis accelerometer, 3 axis gyroscope and 3 axis magnetometer using a q-filter.

Consideration in appendix A are of great interest. I suggest to place them in the main text. Authors should remind the definition of the load factor nmax.
As suggested by the reviewer, we have moved the appendix in the main text of the article and we have revised the text with a clear definition of the load factor.

Appendix C: why not plotting Cl and Cd versus the angle of attack?
We have added these additional graphs in Appendix B.
